# Amphibians and reptiles of the Atlantic Forest in Recôncavo Baiano, east Brazil: Cruz das Almas municipality

**DOI:** 10.3897/zookeys.1060.62982

**Published:** 2021-09-21

**Authors:** Arielson S. Protázio, Airan S. Protázio, Larissa S. Silva, Lennise C. Conceição, Hugo S. N. Braga, Uilton G. Santos, André C. Ribeiro, Amanda C. Almeida, Vívian Gama, Marcos V. S. A. Vieira, Tiago A. F. Silva

**Affiliations:** 1 Centro de Ciências Agrárias, Ambientais e Biológicas, Universidade Federal do Recôncavo da Bahia, Rua Rui Barbosa, Centro, 44380-000, Cruz das Almas, Bahia, Brazil Universidade Federal do Recôncavo da Bahia Cruz das Almas Brazil; 2 Departamento de Ensino, Instituto Federal de Educação, Ciência e Tecnologia da Bahia, Rodovia BA 148, Km 04, Vila Esperança, 44900-000, Irecê, Bahia, Brazil Instituto Federal de Educação Irecê Brazil; 3 Programa de Pós-Graduação em Ecologia e Evolução, Universidade Estadual de Feira de Santana, Av. Transnordestina, S/N, Novo Horizonte, 44936-900, Feira de Santana, Bahia, Brazil Universidade Estadual de Feira de Santana Feira de Santana Brazil; 4 Programa de Pós-Graduação em Zoologia, Universidade Estadual de Santa Cruz, Rodovia Jorge Amado, Km 16, Salobrinho, 45662-900, Ilhéus, Bahia, Brazil Universidade Estadual de Santa Cruz Ilhéus Brazil; 5 Programa de Pós-Graduação em Ciência Animal, Universidade Federal da Bahia, Avenida Adhemar de Barros, S/N, Ondina, 40170-110, Salvador, Bahia, Brazil Universidade Federal da Bahia Salvador Brazil

**Keywords:** Amphisbaena, anuran, diversity, lizards, species richness, snakes, testudines

## Abstract

A list of amphibian and reptile species that occur in open and forested areas of the Atlantic Forest in the municipality of Cruz das Almas, in the Recôncavo Baiano, eastern Brazil is presented. Field sampling occurred between January 2015 to March 2019, totalling 117 samples distributed in three areas: Parque Florestal Mata de Cazuzinha, Mata da Cascalheira, and Riacho do Machado. A total of 1,848 individuals of 69 species (31 anurans, 14 lizards, 19 snakes, two amphisbaenians, and three testudines) was recorded. Additionally, one individual of *Ophiodesstriatus* was found in Mata da Cascalheira after the end of sampling, totalling 15 lizard species and 70 herpetofaunal species. The prevalence of open-area species and the presence of *Phyllopezuslutzae*, *Diploglossuslessonae*, and *Dryadosauranordestina* in interior forest patches are discussed. Additionally, a new record of the invasive terrapin *Trachemysdorbigni* in the State of Bahia is reported.

## Introduction

The Atlantic Forest is a biome that occupies the entire east of South America and of Brazil and is considered one of the most diverse in species richness and levels of endemism (Mittermeier et al. 2004). Regarding amphibians, ca. 625 species occur in this biome, representing more than 50% of the species recorded in Brazil (Haddad et al. 2013; Rossa-Feres et al. 2017; Segalla et al. 2019), while for reptiles, the richness is ca. 312 species, representing 39.2% of the species that occur in the country (Tozetti et al. 2017; Costa and Bérnils 2018). The high species richness in the Atlantic Forest may be associated with a combination of factors such as latitudinal variation (encompassing tropical and subtropical areas), longitudinal variation (with marked variations in rainfall and humidity), elevational variation and biogeographic history, which have shaped different phyto-physiological units and high environmental heterogeneity (Rossa-Feres et al. 2017), reflecting patterns of richness and diversity within well-defined biogeographic units (Vasconcelos et al. 2014; Moura et al. 2017).

Despite its high richness, Brazil is also a country with high levels of threat to biodiversity. According to the Brazil Red Book of Threatened Species of Fauna (ICMBio 2018), 1,173 species are included in some threat category, including 41 species of amphibians and 80 species of reptiles. For the Atlantic Forest, this scenario is extremely worrying. Considered one of the most threatened hotspots in the world (Mittermeier et al. 2004), the biome has 37 endemic anuran species and 39 reptile species included in the list of threatened species (ICMBio 2018). The main reasons for this high rate are the intense activities related to farming and urban growth, which are responsible for causing major changes in the natural landscape (Rodrigues 2005; Silvano and Segalla 2005; ICMBio 2018). According to data from Rezende et al. (2018), only 28% of the Atlantic Forest maintain its original cover, and these areas represent small, isolated forest fragments in a matrix of pasture, plantation or anthropogenic construction; this scenario can have a catastrophic effect on gene flow and biodiversity maintenance.

The State of Bahia is a state with great richness of herpetofauna species (Hamdan and Lira-da-Silva 2012; Dias and Rocha 2014; Freitas et al. 2019). Although in recent years many studies have been developed to characterise the herpetofauna of this state (see Freitas et al. 2016a, b, 2018; Gondim-Silva et al. 2016; Marques et al. 2016; Mira-Mendes et al. 2018; Leite et al. 2019; Souza-Costa et al. 2020; Rojas-Padilla et al. 2020), the vast majority of these initiatives were limited to investigations in specific regions, especially areas of dense ombrophilous forest in the south and southeast of the state (Argôlo 2004; Camurugi et al. 2010; Dias et al. 2014a; Mira-Mendes et al. 2018; Rojas-Padilla et al. 2020; Souza-Costa et al. 2020), sand dune areas on the northern coast of the state (Tinôco et al. 2007; Dias and Rocha 2014; Gondim-Silva et al. 2016; Marques et al. 2016; Napoli et al. 2017) and high-elevation regions of Chapada Diamantina (northern portion of Serra do Espinhaço) (Leite et al. 2008; Xavier and Napoli 2011; Freitas et al. 2012; Magalhães et al. 2015). Allied to this, many of these studies were carried out within protected areas (Leite et al. 2008; Camurugi et al. 2010; Freitas et al. 2012; Garda et al. 2013; Dias et al. 2014a; Magalhães et al. 2015; Mira-Mendes et al. 2018; Rojas-Padilla et al. 2020), highlighting information gaps on the herpetofauna of unprotected areas, making it difficult to identify new areas of ecological relevance.

The Recôncavo Baiano is a region located in the eastern portion of the State of Bahia, corresponding to the portion of land that lies around Todos os Santos Bay (Azevedo 2011). The region has great historical, cultural, and economic importance, standing out for its sugarcane production during the colonial period and, more recently, for its industrial production of petroleum, as well as tobacco and citrus fruits (Sansone 2011). This history of its spatial use was accompanied by intense vegetation suppression and a reduction in original vegetation cover levels. According to data from the Economic-Ecological Zoning of the State of Bahia (Seplan 2015), a management instrument aimed at guiding the use of natural resources, a large part of the Recôncavo Baiano is inserted in the zone called “Tabuleiros Interioranos do Recôncavo” (Interior Trays of the Recôncavo), and only 9.3% of this zone contain the original vegetation cover, although 30% represent a priority area for conservation, revealing alarming levels for biota conservation.

Although a large part of the Recôncavo Baiano is located in the Atlantic Forest, studies characterising the herpetofauna of this region have been conducted almost exclusively in Serra da Jibóia and Serra do Timbó, which represent a set of mountains (elevational range 660–900 m above sea level, respectively) disjoined in the eastern portion of Serra do Espinhaço, in transition with the Caatinga (Juncá 2006; Cruz et al. 2008; Cruz and Napoli 2010; Freitas et al. 2018; Freitas et al. 2019). Furthermore, Freitas (2014) sought to characterise the Atlantic Forest snake fauna of eastern Bahia, including the Recôncavo Baiano; however, despite revealing a high richness of species, his study did not present systematic or standardised searches in the 29 municipalities analysed, which resulted in a sub-sampling in the central portion of the Recôncavo Baiano, including the “Tabuleiros Interioranos”.

This panorama reinforces the appeal for increased studies in forested and open areas of eastern Bahia to improve the characterisation of the richness and species composition of the herpetofauna of this portion of the Atlantic Forest. This information is essential to identify and monitor population fluctuations, enabling an accurate diagnosis of the ecosystem’s integrity and allowing access to the mechanisms that are involved in generating the region’s fauna diversity. In this study, we present a list of amphibian and reptile species that occur in open and forested areas of the municipality of Cruz das Almas, as part of a long-term project that seeks to characterise the herpetofauna of all the municipalities that are part of the Recôncavo Baiano, to minimise differences in the sample efforts along the different regions of the Atlantic Forest and to fill the information gaps regarding the state fauna.

## Materials and methods

### Study area

**Figure 1.**
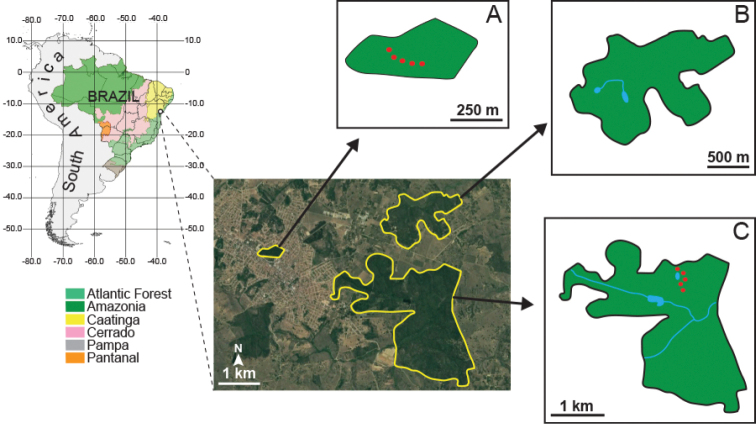
Geographical location of the Cruz das Almas municipality and areas of study **A** Parque Florestal Mata de Cazuzinha **B** Mata da Cascalheira **C** Riacho do Machado. Red dots represent localisation and disposition of the pitfall traps.

The study was conducted in Cruz das Almas municipality, Bahia State, in northeast Brazil (12°40'25"S, 39°06'05"W) (Figure 1). Cruz das Almas is located in the eastern portion of Bahia State and inserted in the region of the Recôncavo Baiano, in the Atlantic Forest biome. The region is characterised by original vegetation of Semideciduous Seasonal Forest (Brazão and Araújo 1981); however, it has been almost completely replaced by grazing areas for cattle raising and by plantations. According to the Köppen classification, the climate is tropical monsoon (Am), with an average temperature of 23.9 °C and an annual rainfall of 1,131.2 mm (Silva et al. 2016). Cruz das Almas is located in the area “Tabuleiros Interioranos do Recôncavo” (Interior Trays of the Recôncavo), characterised by a flat and gently undulating top relief, not exceeding 200 m in elevation, in addition to a large amount of micro-basins, which is propitious for agricultural production. Only 5% of the “Tabuleiros Interioranos do Recôncavo” area are inserted in some conservation units, all of them of sustainable use (Seplan 2015).

**Figure 2.**
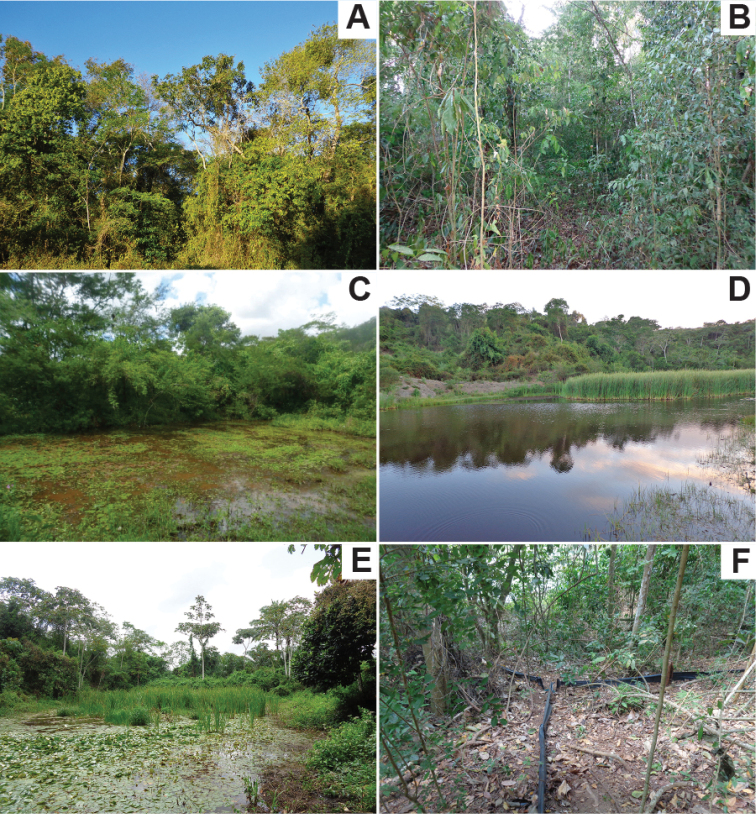
Habitats sampled during the field works **A** view of Mata de Cazuzinha showing tall trees and shrubs **B** inside the Mata de Cazuzinha **C** temporary pond in Mata da Cascalheira **D** lagoon in Mata da Cascalheira **E** temporary pond in the Riacho do Machado **F** pitfall trap in the interior of the forest at Riacho do Machado.

Field activities were concentrated in three areas of the municipality that presented good conservation status and potential for finding herpetofauna specimens (Figure 2):

(i) Parque Florestal Mata de Cazuzinha (12°39'58"S, 39°06'25"W; elevation 235 m): This is a forest fragment of ca. 20 ha, inserted in an urban matrix. It is considered an area of secondary vegetation, but presents a homogeneous and dense aspect, with predominance of arboreal vegetation of medium and large size. There is no evidence of permanent waterbodies inside the Cazuzinha forest, yet it has a forest structure that enables moisture concentration. Because it is located in an urban environment, the Cazuzinha forest suffers great anthropic pressure associated with hunting, wood removal and waste deposit. The area is intensely frequented by the local population for leisure activities.

(ii) Riacho do Machado (12°40'35.89"S, 39°25'59"W; elevation 226 m): The largest forest patch fragment in Cruz das Almas municipality. It is located between the experimental area of the Empresa Brasileira de Pesquisas Agropecuárias (Brazilian Agricultural Research Corporation) and the campus of the Federal University of Recôncavo da Bahia. This area is inserted in a hillside region and has medium-sized trees. It has a central lake ca. 5 m deep and a stream that names the patch. The homogeneous aspect of the vegetation indicates that the area has not been much accessed for wood removal, probably due to the difficulty of access. Nevertheless, during this study, some actions of burning, cutting and timber extraction were witnessed. Inside the forest, there are regions of “swamps”, which are propitious environments for the reproduction of anurans, as well as regions with rocky outcrops.

(iii) Mata da Cascalheira (12°39'29"S, 39°04'48"W; elevation 212 m): Cascalheira forest patch is an area of secondary forest, with predominance of shrubby vegetation and grasses. The forest patch is inserted in a region of hillsides and has a lagoon in the central region, which is ca. 4 m deep and contains large amounts of cattail and macrophytes. Mata da Cascalheira is inserted in the campus of the Federal University of Recôncavo da Bahia, which is a benchmark in agricultural studies. Thus, much of the patch has already been used as pastureland or arable land, with the formation of small puddles during rain events. In addition, it is possible to find some houses near the forest patch, where the residents engage in subsistence farming activity. Some areas of the Mata da Cascalheira were used for the extraction of land and stones for construction.

### Data collection

We conducted the field activities from January 2015 to Mach 2019, through non-standard day and night collections in the three different areas, totalling 117 samples. All daytime collections started at 8 am and ended at 5 pm, while night-time collections started at 6 pm and ended at midnight. The collections were performed by at least three and at most eight researchers. To collect the specimens, we used the techniques of visual encountering through random search inside and around the patches, aided by shot guns, and the places investigated were holes, burrows, tree trunks, fallen trunks, the interior of bromeliads, rocks, and all microhabitats conducive to the encounter of individuals in shelter or in activity (Figure 2A–E). For amphibians, we also used the acoustic search to find males in vocalisation activity, concentrating the activities around the waterbodies (Heyer et al. 1994). To enhance the encounter of herpetofauna specimens, we installed five pitfall traps in each area. The traps were arranged in a Y-shape, with four 30-L buckets connected by three 8-m drift fences build with plastic sheets (Figure 2F). The stations were ca. 60 meters apart and remained installed for 32 days in each area; they were inspected daily. The pitfall traps were not efficiently implemented in the Mata da Cascalheira due to the strong human presence, with consequent damage to the buckets, which made it impossible to use the traps on site. Nevertheless, as we did not seek to perform a comparative analysis between the areas but rather to summarise the species of the herpetofauna found in the region, this scenario did not interfere with our objectives. In addition, we also employed the techniques of occasional encounters and encounters by third parties to better characterise the herpetofauna.

All animals collected were euthanised via intraperitoneal injection of 2% lidocaine, fixed in 10% formaldehyde, preserved in 70% alcohol, and deposited in the Herpetological Collection of the Universidade Federal do Recôncavo da Bahia (Sisbio Permit 46558-1 and 46558-2; CEUA-UFRB Permit 23007.007559/2016-71). The animals collected had a small fragment of the liver extracted to create a genetic database of the herpetofauna of the Recôncavo Baiano region, providing support for future studies.

### Analyses

To evaluate the quality of our sampling effort, we used the data of species richness and abundance of individuals to produce rarefaction curves (1,000 randomisations), using the ESTIMATES 9.1.0 program (Colwell 2013). Since our samples from non-standard samples, we used the individual based curve to standardise our effort, as each sample unit is an individual. Subsequently, the observed richness was compared with the estimated richness from the non-parametric estimators Bootstrap, Chao 2 and Jackknife 1 and 2 (Magurran 2004). As these estimators are sensitive to singletons and doubletons, species with low abundance were inserted in analyses. We built a rarefaction curve for amphibians, a rarefaction curve for lizards, a rarefaction curve for snakes and a rarefaction curve joining all groups (herpetofauna), including amphisbaenians and testudines. In addition, we verified which species are typical of the Atlantic Forest, based on Rossa-Feres et al. (2017) and Tozetti et al. (2017), for amphibians and reptiles, respectively.

Finally, we compared the similarity of the species composition of the amphibian and reptiles of Cruz das Almas with the species composition of other assemblages of the Brazilian Atlantic Forest. We performed an analysis considering only anurans, lizards, and snakes. Data on the composition of anurans from other assemblages were obtained from 32 studies, while data on the composition of lizards and snakes were obtained from 21 studies. For this, we subdivided the Atlantic Forest into four biogeographic sub-regions, based on Silva and Casteleti (2003) to identify regional similarities: (1) north of the São Francisco River, covering the states of Paraíba (PB), Pernambuco (PE), and Rio Grande do Norte (RN) – Mata do Buraquinho (Santana et al. 2008); Macaíba (Magalhães et al. 2013); Boca da Mata (Palmeira and Gonçalves 2015); Serra do Urubu (Roberto et al. 2017); Reserva Biológica (REBIO) Guaribas (Mesquita et al. 2018); (2) south of the São Francisco River, covering the states of Bahia (BA) and Sergipe (SE) – Mata de São João (Couto-Ferreira et al. 2011; Marques et al. 2011); Mata do Junco (Morato et al. 2011); Jequié (Lantyer-Silva et al. 2013); Serra Bonita (Dias et al. 2014a); Conde (Gondim-Silva et al. 2016); Serra da Jibóia (Freitas et al. 2018); Reserva Michelin (Mira-Mendes et al. 2018); Serra do Timbó (Freitas et al. 2019); Serra das Lontras (Rojas-Padilla et al. 2020); Serra Azul and Serra de Mandim (Souza-Costa et al. 2020); (3) southeast Brazil and the Serra do Mar region, covering the states of Espírito Santo (ES), Minas Gerais (MG), São Paulo (SP), and Rio de Janeiro (RJ) – Rio Novo (Feio and Ferreira 2005); Reserva Florestal (RF) de Morro Grande (Dixo and Verdade 2006); Rio Claro (Zina et al. 2007); Estação Ambiental (EA) de Peti (Bertoluci et al. 2009); Ilha de Anchieta (Cicchi et al. 2009); Parque Estadual (PE) Jurupará (Condez et al. 2009); Estação Ecológica (EE) Juréia-Itatins (Narvaes et al. 2009); Parque Estadual Turístico (PET) do Alto Ribeira (Araujo et al. 2010); Parque Estadual (PE) Carlos Botelho (Forlani et al. 2010); Alto Rio Muriaé (Santana et al. 2010); Estação Ecológica (EE) do Paraíso (Vrcibradic et al. 2011); Serra do Brigadeiro (Moura et al. 2012); Parque Natural Municipal (PNM) de Grumari (Telles et al. 2012); Reserva Ecológica (RE) de Guapiaçu (Almeida-Gomes et al. 2014); São Roque do Canaã (Mônico et al. 2017); and (4) – south Brazil and the Araucaria Forest region, covering the states of Paraná (PR), Santa Cararina (SC) and Rio Grande do Sul (RS) – Rio Grande (Quintela et al. 2006); Parque Estadual (PE) de Itapeva (Colombo et al. 2008); Morretes (Armstrong and Conte 2010); Parque Nacional (PN) das Araucárias (Lucas and Marocco 2011); Parque Natural Municipal (PNM) de Sertão (Zanella et al. 2013). We excluded the species identified at the level of genus and those in which the authors had doubts about the specific epithet. Cluster analysis was performed using the UPGMA algorithm and Jaccard index in the Past 4.05 program (Hammer et al. 2001).

## Results

**Figure 3.**
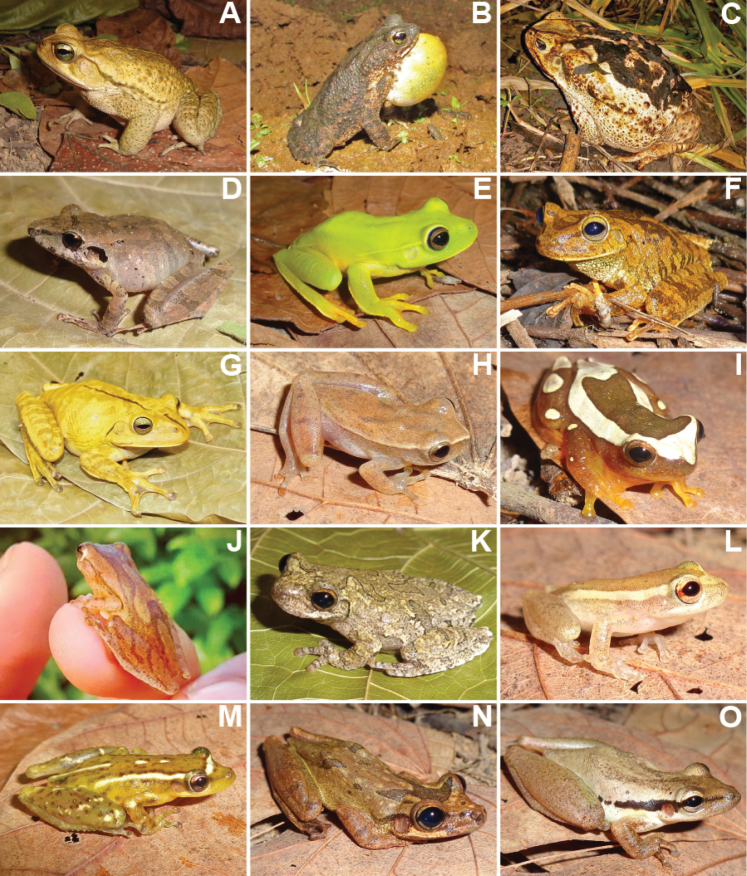
Anuran species identified at Cruz das Almas municipality, Bahia State **A***Rhinellacrucifer***B***Rhinellagranulosa***C***Rhinellajimi***D***Pristimantispaulodutrai***E***Boanaalbomarginata***F***Boanacrepitans***G***Boanafaber***H***Dendropsophusbranneri***I***Dendropsophuselegans***J***Dendropsophusminutus***K***Dendropsophusnovaisi***L***Dendropsophusoliveirai***M***Scinaxauratus***N***Scinaxeurydice***O***Scinaxpachycrus*.

**Figure 4.**
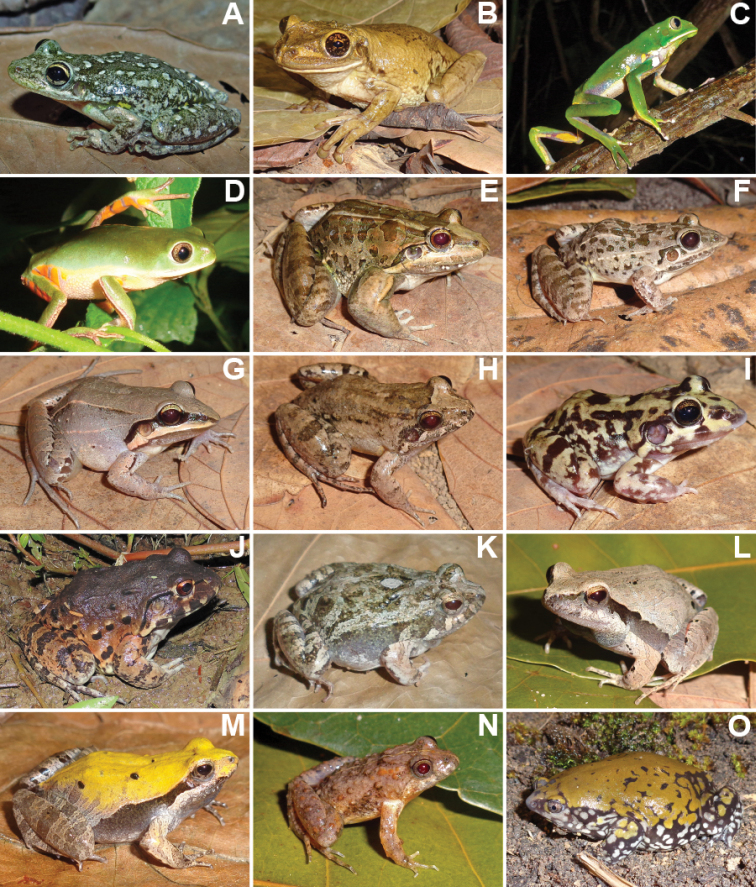
Anuran species identified at Cruz das Almas municipality, Bahia State **A***Scinaxx-signatus***B***Trachycephalusatlas***C***Phyllomedusabahiana***D***Pithecopusnordestinus***E***Leptodactylusmacrosternum***F***Leptodactylusfuscus***G***Leptodactylusmystaceus***H***Leptodactylusnatalensis***I***Leptodactylustroglodytes***J***Leptodactylusvastus***K***Physalaemusalbifrons***L***Physalaemuscuvieri***M***Physalaemuskroyeri***N***Pseudopaludicolaflorencei***O***Dermatonotusmuelleri*.

**Figure 5.**
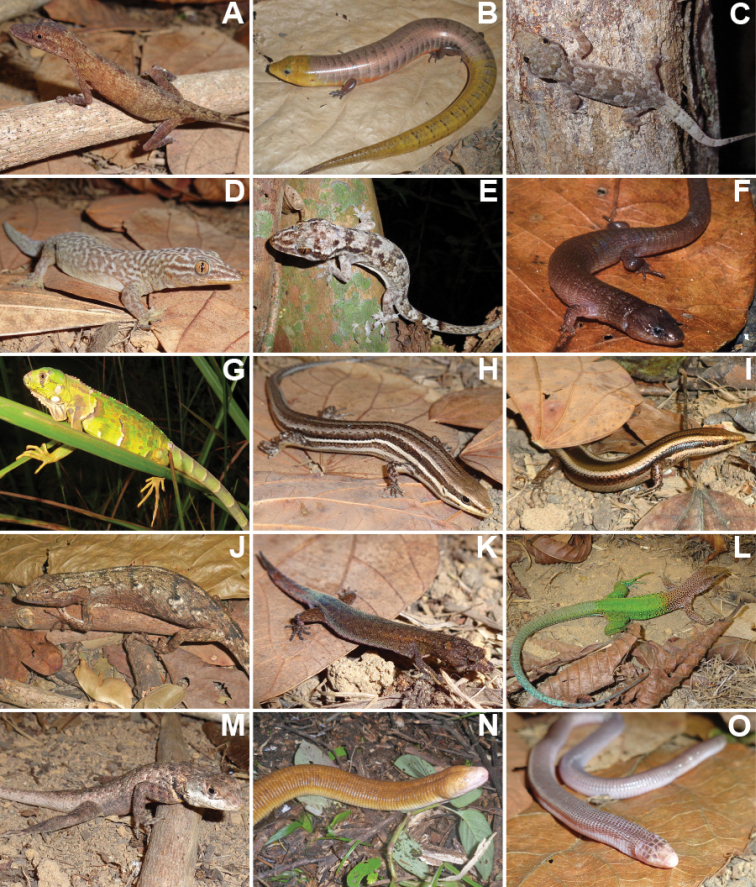
Reptile species identified at Cruz das Almas municipality, Bahia State **A***Noropsfuscoauratus***B***Diploglossuslessonae***C***Hemidactylusmabouia***D***Phyllopezuslutzae***E***Phyllopezuspollicaris***F***Dryadosauranordestina***G***Iguanaiguana***H***Brasiliscincusheathi***I***Psychosauramacrorhyncha***J***Polychrusacutirostris***K***Coleodactylusmeridionalis***L***Ameivaameiva***M***Tropidurushispidus***N***Amphisbaenaalba***O***Amphisbaenavermicularis*.

**Figure 6.**
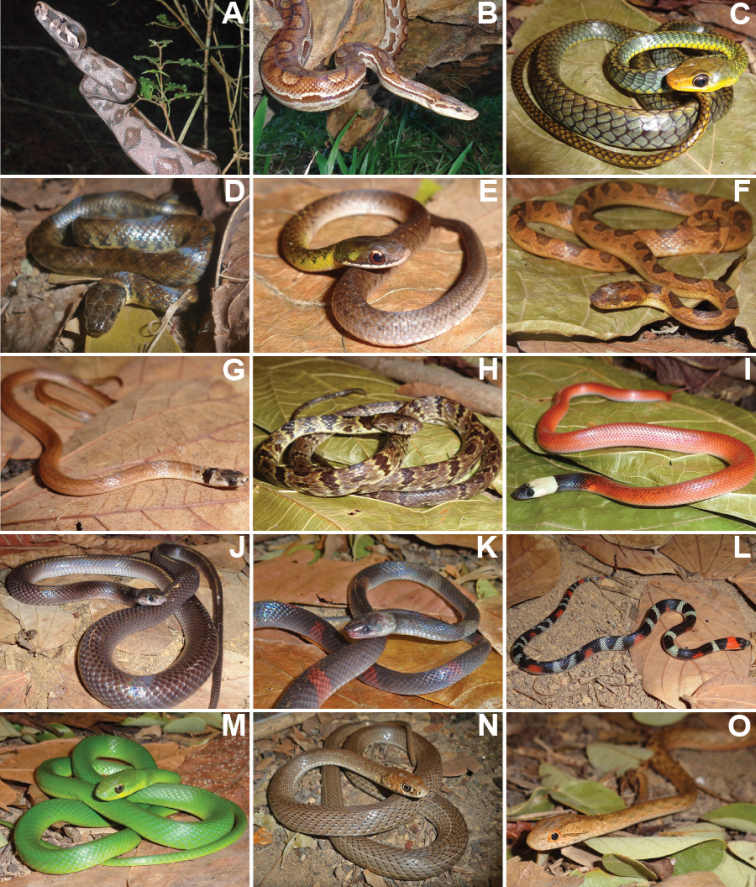
Reptile species identified at Cruz das Almas municipality, Bahia State **A***Boaconstrictor***B***Epicratesassisi***C***Chironiuscarinatus***D***Erytrolamprusmiliaris***E***Erytrolamprusreginae* (juvenile) **F***Leptodeiraannulata***G***Tantillamelanocephala***H***Dipsasneuwiedi***I***Pseudoboanigra* (juvenile) **J***Pseudoboanigra* (adult) **K***Oxyrhopuspetolarius***L***Oxyrhopustrigeminus***M***Philodryasolfersii***N***Philodryaspatagoniensis***O***Thamnodynastespallidus*.

**Figure 7.**
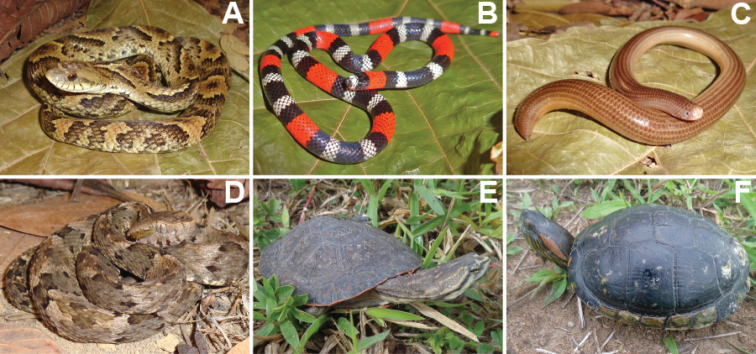
Reptile species identified at Cruz das Almas municipality, Bahia State **A***Xenodonmerremii***B***Micrurusibiboboca***C***Amerotyphlopsbrongersmianus***D***Bothropsleucurus***E***Phrynopsgeoffroanus***F***Trachemysdorbigni*.

We recorded a total of 1,848 individuals, distributed in 69 species of amphibians and reptiles (31 anurans, 14 lizards, 19 snakes, two amphisbaenians, and three testudines). Additionally, in December 2020, after the end sampling, we found an individual of *Ophiodesstriatus* in Mata da Cascalheira, adding a lizard species to the list, totalling 15 lizard species and 70 herpetofauna species. The anurans identified belong to the families Bufonidae (3 spp.), Craugastoridae (1 sp.), Hylidae (13 spp.), Leptodactylidae (11 spp.), Microhylidae (1 sp.) and Phyllomedusidae (2 spp.) (Table 1, Figures 3, 4). The 15 species of lizards belong to the families Dactyloidae (1 sp.), Diploglossidae (2 spp.), Gekkonidae (3 spp.), Gymnophthalmidae (1 sp.), Iguanidae (1 sp.), Mabuyidae (2 spp.), Polychrotidae (1 sp.), Sphaerodactylidae (1 sp.), Teiidae (2 spp.) and Tropiduridae (1 sp.). The 19 snake species belong to the families Boidae (2 spp.), Colubridae (5 spp.), Dipsadidae (9 spp.), Elapidae (1 sp.), Typhlopidae (1 sp.) and Viperidae (1 sp.). The two amphisbaenians species belong to the family Amphisbaenidae (2 spp.), and the testudines belong to the families Chelidae (1 sp.), Emydidae (1 sp.) and Testudinidae (1 sp.) (Table 2, Figures 5–7).

**Table 1.** Check list of amphibians identified at Cruz das Almas municipality, Bahia State.

Taxon	Species	Abundance
** Anura **		
Bufonidae	*Rhinellacrucifer* (Wied-Neuwied, 1821)	2
	*Rhinellagranulosa* (Spix, 1824)	24
	*Rhinellajimi* (Stevaux, 2002)	54
Craugastoridae	*Pristimantispaulodutrai* (Bokermann, 1975)	118
Hylidae	*Boanaalbomarginata* (Spix, 1824)	107
	*Boanacrepitans* (Wied-Neuwied, 1824)	29
	*Boanafaber* (Wied-Neuwied, 1821)	5
	*Dendropsophuselegans* (Wied-Neuwied, 1824)	229
	*Dendropsophusbranneri* (Cochran, 1948)	84
	*Dendropsophusminutus* (Peters, 1872)	7
	*Dendropsophusnovaisi* (Bokermann, 1968)	11
	*Dendropsophusoliveirai* (Bokermann, 1963)	63
	*Scinaxauratus* (Wied-Neuwied, 1821)	46
	*Scinaxeurydice* (Bokermann, 1968)	23
	*Scinaxpachycrus* (Miranda-Ribeiro, 1937)	3
	*Scinaxx-signatus* (Spix, 1824)	60
	*Trachycephalusatlas* Bokermann, 1966	2
Leptodactylidae	*Leptodactylusfuscus* (Schneider, 1799)	38
	*Leptodactylusmacrosternum* Miranda-Ribeiro, 1926	100
	*Leptodactylusmystaceus* (Spix, 1824)	16
	*Leptodactylusnatalensi*s Lutz, 1930	24
	*Leptodactylusvastus* Lutz, 1930	24
	*Leptodactylustroglodytes* Lutz, 1926	54
	*Physalaemusalbifrons* (Spix, 1824)	1
	*Physalaemuscuvieri* Fitzinger, 1826	106
	*Physalaemuskroyeri* (Reinhardt & Lütken, 1862)	95
	Pseudopaludicolacf.mystacalis (Cope, 1887)	1
	*Pseudopaludicolaflorencei* Andrade, Haga, Lyra, Leite, Kwet, Haddad, Toledo & Giaretta, 2018	16
Microhylidae	*Dermatonotusmuelleri* (Boettger, 1885)	10
Phyllomedusidae	*Phyllomedusabahiana* Lutz, 1925	13
	*Pithecopusnordestinus* (Caramaschi, 2006)	30

**Table 2.** Check list of reptiles identified at Cruz das Almas municipality, Bahia State.

Taxon	Species	Abundance
** Squamata **		
Amphisbaenidae	*Amphisbaenaalba* Linnaeus, 1758	14
	*Amphisbaenavermicularis* Wagler, 1824	5
**Lizards**		
Dactyloidae	*Noropsfuscoauratus* (D’Orbigny, 1837)	9
Diploglossidae	*Diploglossuslessonae* Peracca, 1890	2
	*Ophiodesstriatus* (Spix, 1824)	1
Gekkonidae	*Hemidactylusmabouia* (Moreau de Jonnès, 1818)	144
	*Phyllopezuslutzae* (Loveridge, 1941)	11
	*Phyllopezuspollicaris* (Spix, 1825)	50
Gymnophthalmidae	*Dryadosauranordestina* Rodrigues, Freire, Pellegrino & Sites, 2005	1
Iguanidae	*Iguanaiguana* (Linnaeus, 1758)	1
Mabuyidae	*Brasiliscincusheathi* (Schmidt & Inger, 1951)	5
	*Psychosauramacrorhyncha* (Hoge, 1946)	2
Polychrotidae	*Polychrusacutirostris* Spix, 1825	3
Sphaerodactylidae	*Coleodactylusmeridionalis* (Boulenger, 1888)	19
Teiidae	*Ameivaameiva* (Linnaeus, 1758	35
	*Salvatormerianae* Duméril & Bibron, 1839	4
Tropiduridae	*Tropidurushispidus* (Spix, 1825)	72
**Snakes**		
Boidae	*Boaconstrictor* Linnaeus, 1758	4
	*Epicratesassisi* Machado, 1945	5
Colubridae	*Chironiuscarinatus* (Linnaeus, 1758)	1
	*Erythrolamprusmiliaris* (Linnaeus, 1758)	1
	*Erythrolamprusreginae* (Linnaeus, 1758)	3
	*Leptodeiraannulata* (Linnaeus, 1758)	1
	*Tantillamelanocephala* (Linnaeus, 1758)	2
Dipsadidae	*Dipsasneuwiedi* (Ihering, 1911)	3
	*Helicopsleopardinus* (Schlegel, 1837)	1
	*Pseudoboanigra* (Duméril, Bibron & Duméril, 1854)	5
	*Oxyrhopuspetolarius* (Linnaeus, 1758)	1
	*Oxyrhopustrigeminus* Duméril, Bibron & Duméril, 1854	6
	*Philodryasolfersii* (Lichtenstein, 1823)	7
	*Philodryaspatagoniensis* (Girard, 1858)	1
	*Thamnodynastespallidus* (Linnaeus, 1758)	3
	*Xenodonmerremii* (Wagler, 1824)	3
Elapidae	*Micrurusibiboboca* (Merrem, 1820)	17
Typhlopidae	*Amerotyphlopsbrongersmianus* (Vanzolini, 1976)	3
Viperidae	*Bothropsleucurus* Wagler, 1824	6
** Testudines **		
Chelidae	*Phrynopsgeoffroanus* (Schweigger, 1812)	1
Emydidae	*Trachemysdorbigni* (Duméril & Bibron, 1835)	1
Testudinidae	*Chelonoidiscarbonarius* (Spix, 1824)	1

**Figure 8.**
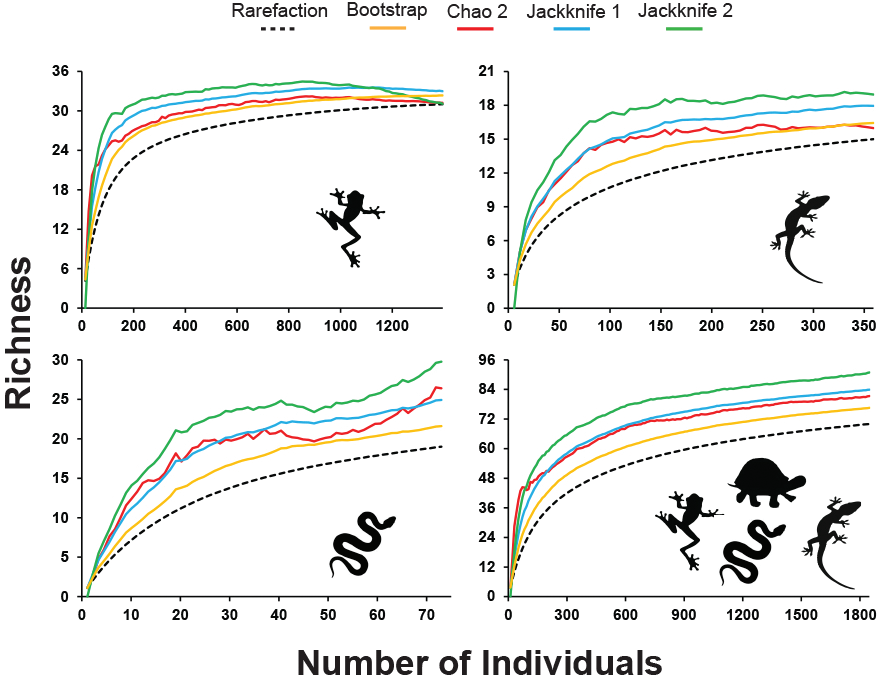
Species rarefaction curves and richness estimators for amphibians, lizards, snakes and herpetofauna collected at Cruz das Almas municipality. Herpetofauna represent the combination of amphibians, lizards, snakes, amphisbaenians, and testudines.

The rarefaction curve approached the asymptote only for amphibians, demonstrating that the sample effort managed to obtain a satisfactory representation of species (Figure 8). However, richness estimators predicted the existence of species not yet added to the list, ranging from one to two species of anurans. For lizards, snakes, and herpetofauna, the rarefaction curves did not reach the asymptote, and the richness estimators added between one and four species, between three and 11 species, and between seven and 21 species, respectively (Table 3). The observation of the species composition of the anurans assemblage from Cruz das Almas revealed the presence of two groups of species: species endemic to the Atlantic Forest (*Rhinellacrucifer*, *Pristimantispaulodutrai*, *Boanaalbomarginata*, *Dendropsophusbranneri*, *Dendropsophuselegans*, *Dendropsophusnovaisi*, *Phyllomedusabahiana*, *Scinaxauratus*, *Scinaxeurydice*) and species distributed in two or more biomes (*Rhinellagranulosa*, *Rhinellajimi*, *Boanacrepitans*, *Boanafaber*, *Dendropsophusminutus*, *Dendropsophusoliveirai*, *Scinaxx-signatus*, *Scinaxpachycrus*, *Trachycephalusatlas*, *Pithecopusnordestinus*, *Leptodactylusfuscus*, *Leptodactylusmacrosternum*, *Leptodactylusmystaceus*, *Leptodactylusnatalensis*, *Leptodactylustroglodytes*, *Leptodactylusvastus*, *Physalaemusalbifrons*, *Physalaemuscuvieri*, *Physalaemuskroyeri*, Pseudopaludicolacf.mystacalis, *Pseudopaludicolaflorencei*, *Dermatonotusmuelleri*). For lizards, only *Phyllopezuslutzae* and *Dryadosauranordestina* are endemic to the Atlantic forest, while for snakes only *Bothropsleucurus* is endemic.

**Table 3.** Mean and standard deviation of the species richness estimated with different estimators in Cruz das Almas municipality. Herpetofauna represent the combination of amphibians, lizards, snakes, amphisbaenians, and testudines.

	**Amphibians**	**Lizards**	**Snakes**	**Herpetofauna**
Observed richness	31	15	19	70
Bootstrap	32 ± 0	16 ± 0	22 ± 0	77 ± 0
Chao 2	31 ± 0.62	16 ± 1.79	26 ± 8.06	81 ± 8.05
Jackknife 1	33 ± 1.39	18 ± 1.68	25 ± 2.32	84 ± 3.58
Jackknife 2	31 ± 0	19 ± 0	30 ± 0	91 ± 0
Singletons	2	3	6	14

**Figure 9.**
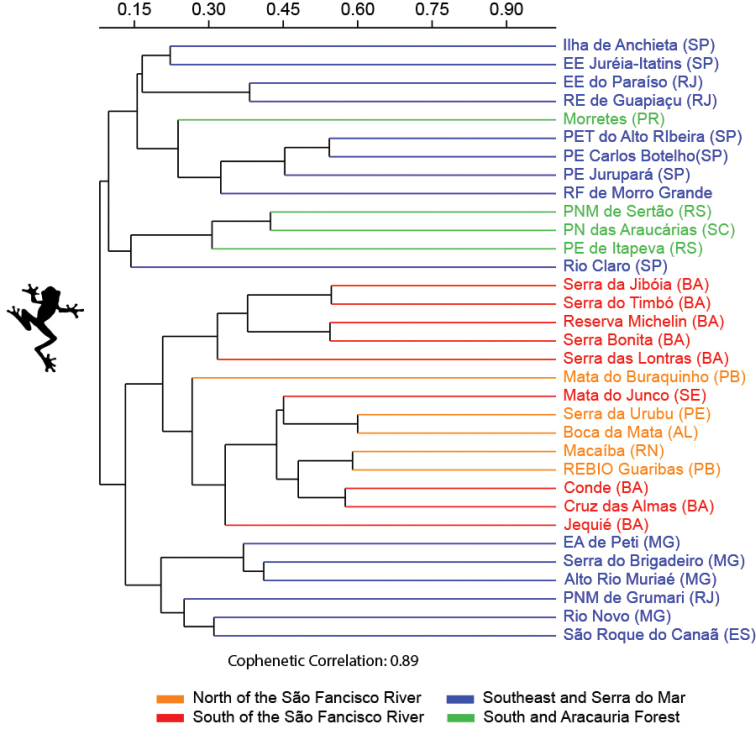
Dendrogram of cluster analysis (Jaccard Indices) of the anuran species composition from 33 localities in Brazilian Atlantic Forest. Abbreviations in Materials and methods.

**Figure 10.**
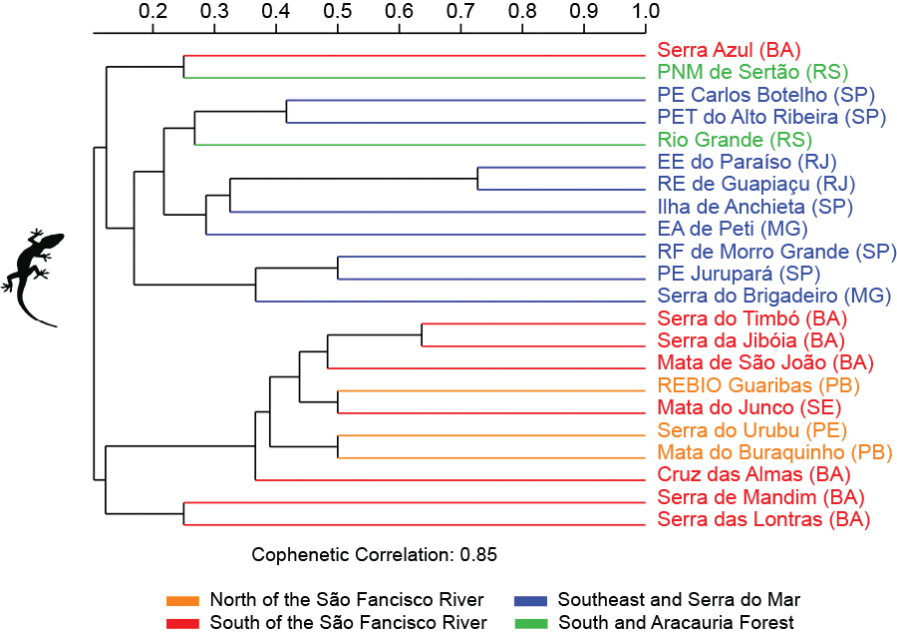
Dendrogram of cluster analysis (Jaccard Indices) of the lizard species composition from 22 localities in Brazilian Atlantic Forest. Abbreviations in Materials and methods.

**Figure 11.**
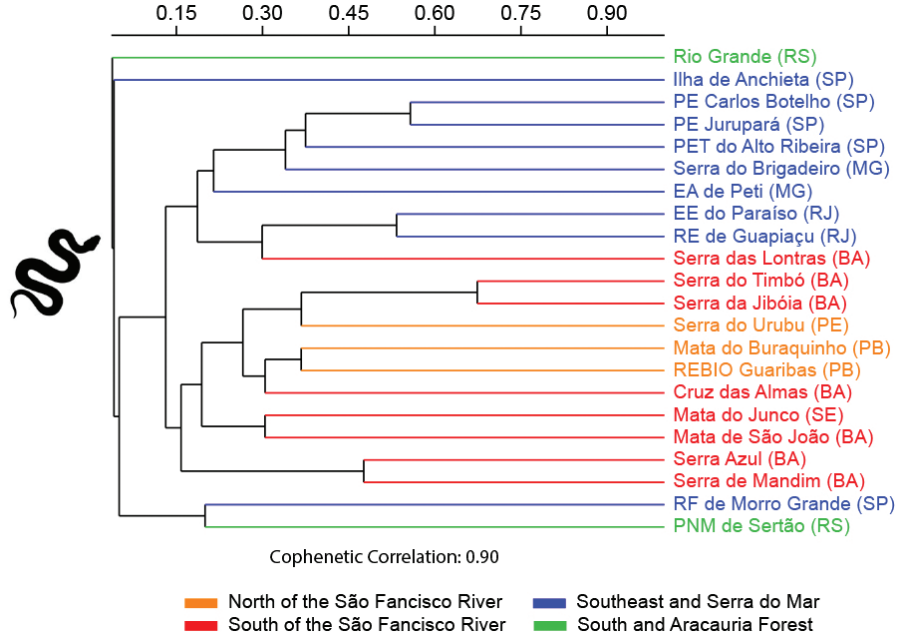
Dendrogram of cluster analysis (Jaccard Indices) of the snake species composition from 22 localities in Brazilian Atlantic Forest. Abbreviations in Materials and methods.

The cluster analysis revealed that the assemblages of anurans, lizards, and snakes from Cruz das Almas formed close groups with those assemblages from northeast Brazil, specifically from north and south of the São Francisco River. This result indicates that there is a faunal similarity between assemblages inserted in the Central Corridor of the Atlantic Forest and the Pernambuco Endemism Centre (Figures 9–11). The anuran assemblage was more similar to the Conde assemblage, whereas the snake assemblage was more similar to the Mata do Buraquinho and REBIO Guaribas assemblages. For lizards, the Cruz das Almas assemblage was more similar to the Serra do Timbó, Serra da Jibóia, Mata de São João, REBIO Guaribas, Mata do Junco, Serra do Urubu, and Mata do Buraquinho assemblages.

## Discussion

The anuran species richness identified in Cruz das Almas corresponds to 5% of the anuran richness currently known for the Atlantic Forest (Rossa-Feres et al. 2017) and 2.8% of the anuran richness of Brazil (Segalla et al. 2019). Furthermore, 29% of the anuran species identified in the Cruz das Almas assemblage are endemic to the Atlantic Forest (Rossa-Feres et al. 2017). The richness of Cruz das Almas anurans, when compared to other assemblages in the Atlantic Forest, revealed a value similar to those from the north and the south of the São Francisco River (Morato et al. 2011, *n* = 33; Lantyer-Silva et al. 2013, *n* = 31; Magalhães et al. 2013, *n* = 34; Palmeira and Gonçalves 2015, *n* = 32; Mesquita et al. 2018, *n* = 34) and higher than some assemblages from south and southeast Brazil (Feio and Ferreira 2005, *n* = 20; Zina et al. 2007, *n* = 24; Narvaes et al. 2009, *n* = 20; Zanella et al. 2013, *n* = 23). However, it was a lower value than those from other assemblages from southeast Brazil (Araujo et al. 2010, *n* = 58; Forlani et al. 2010, *n* = 64; Moura et al. 2012, *n* = 57; Almeida-Gomes et al. 2014, *n* = 70) and from the north of the São Francisco River (Roberto et al. 2017, *n* = 46). Within Bahia State, the richness of Cruz das Almas revealed a similar value to the assemblages from the southeast (Dias et al. 2014b, *n* = 33–40) and the northern coast (Juncá 2006, *n* = 25; Bastazini et al. 2007, *n* = 30; Gondim-Silva et al. 2016, *n* = 33). Conversely, this value was low when compared with some southern (Dias et al. 2014a, *n* = 79; Rojas-Padilla et al. 2020, *n* = 49) and southeast (Mira-Mendes et al. 2018, *n* = 68) assemblages of the state.

According to Lantyer-Silva et al. (2013), locations within the Atlantic Forest where the richness of amphibians ranging around 32 species can be considered as having an intermediate richness, which seems to be a common pattern for assemblages of Atlantic Forest-Caatinga ecotones. These assemblages are characterised by higher species richness than the Caatinga, as well as a species composition common to both biomes (Lantyer-Silva et al. 2013). Thus, the richness and composition of species of the anurans assemblage from Cruz das Almas leads to a fauna characteristic of transition zones between the Atlantic Forest and Caatinga biomes, even though Cruz das Almas municipality is inserted into the Atlantic Forest biome. This scenario goes against our initial expectations. We expected that the Cruz das Almas assemblage would reveal a greater quantity of endemic species of the Atlantic Forest. However, the species composition was dominated by generalist species, typically associated with open lands. We did not rule out the possibility that the species composition observed in this study is associated with the history of intense land use, the accentuated suppression of vegetation and changes in the natural landscape of Cruz das Almas, which reflected the formation of secondary forest patches, as well as intense open areas destined for pasture and plantation. This remarkable anthropisation may have promoted the selection of species more tolerant to landscape changes, as well as the extinction of species more specialised in forest habitats, causing a reduction in richness (Almeida-Gomes and Rocha 2014). However, studies that seek to verify the variation in species composition between areas with different levels of conservation in this region could better clarify this assumption.

None of the anuran species found in our study revealed an unusual or unexpected record for the region, having been previously reported for the State of Bahia and the Atlantic Forest (Dias et al. 2014a; Gondim-Silva et al. 2016; Freitas et al. 2018; Mira-Mendes et al. 2018; Freitas et al. 2019). Nevertheless, we report a new record of the recently described *Pseudopaludicolaflorencei* for Bahia State. Besides the locality type (Andaraí, Bahia), the species has been recognised in only two localities (Mutuípe, in the State of Bahia and Nanuque, in the State of Minas Gerais) (Andrade et al. 2018). In Cruz das Almas, *P.florencei* was observed vocalising in small puddles or fillets of water formed after the first rains, always in open areas near Riacho do Machado and Mata da Cascalheira. The species was identified on the basis of morphological and acoustic characteristics, consistent with the original description. Thus, we believe that a more accurate analysis of the advertisement call of other individuals of the genus *Pseudopaludicola* can reveal the presence of the species in other localities of the Atlantic Forest of the east of Bahia.

The Cruz das Almas assemblage presented a low number of typical species from forested areas and prevalence of typical species from open land. This result explains the greater similarity of the Cruz das Almas assemblage with other assemblage from open land and Atlantic Forest-Caatinga ecotones observed in our cluster analysis. Anthropic actions may have shaped the current pattern of the Cruz das Almas assemblage, leading to the reduction of species more specialised to forest habitats. This scenario reveals the need for greater efforts to preserve the remaining forest fragments in the region. Nevertheless, *Pristimantispaulodutrai* was the dominant species in the interior of forest fragments in Mata de Cazuzinha, often found vocalising perched on herbaceous vegetation. Besides *P.paulodutrai*, only *Rhinellajimi*, *Leptodactylustroglodytes*, *Physalaemuscuvieri*, and *Physalaemuskroyeri* were also identified in the Mata de Cazuzinha, but only on the edge, where they used waterbodies formed after the rains. We believe that *P.paulodutrai’s* dominance of the urban forest fragment is due to the absence of waterbodies inside the fragment, which may have limited the permanence of other species.

The reptile species richness of the Cruz das Almas assemblage corresponds to 12.5% of the known reptile richness for the Atlantic Forest (Tozetti et al. 2017) and to 4.9% of the known species richness for Brazil (Costa and Bérnils 2018). Considering the taxa individually, for the Atlantic Forest, the lizards richness corresponds to 17.9%, that of snakes to 10%, that of amphisbaenians to 9.1% and that of testudines to 21.4% (except for *Trachemysdorbigni*) of the biome richness, while for Brazil, the lizard richness corresponds to 5.4%, that of snakes to 4.7%, that of amphibians to 2.8% and that of testudines to 5.5% (Tozetti et al. 2017; Costa and Bérnils 2018). Attempts to compare the richness of reptiles identified in the Cruz das Almas assemblages with the richness of other assemblages of the Atlantic Forest of the Bahia State were hampered due to the lack of systematic inventories involving the different taxonomic categories. We noticed the presence of robust information for snakes, while the information was less common for lizards, evidencing the need for greater efforts to characterise the species of the group in the several phyto-physiognomies of the Atlantic Forest of the State.

For the lizards, our results revealed the presence of species previously recorded for Bahia State and the Atlantic Forest (Couto-Ferreira et al. 2011; Hamdan and Lira-da-Silva 2012; Freitas 2014). However, a comparison of the assemblage richness of Cruz das Almas lizards with that of other assemblages from the Atlantic Forest revealed a value similar to that of assemblages in the north and south of the São Francisco River (Santana et al. 2008, *n* = 13; Roberto et al. 2017, *n* = 16), while the value was higher in relation to several assemblages in the south and southeast of Brazil (Dixo and Verdade 2006, *n* = 5; Quintela et al. 2006, *n* = 8; Bertoluci et al. 2009, *n* = 5; Forlani et al. 2010, *n* = 10; Moura et al. 2012, *n* = 9; Almeida-Gomes et al. 2014, *n* = 10) and lower in relation to one assemblage north of the São Francisco River (Mesquita et al. 2018, *n* = 20). Observing the richness of only the assemblages within the State of Bahia, the richness found in the present study was inferior to the richness of lizards from the northern coast (Couto-Ferreira et al. 2011, *n* = 23), Serra da Jibóia (Freitas et al. 2018, *n* = 19), and Serra do Timbó (Freitas et al. 2019, *n* = 19). However, the richness of Cruz das Almas lizards was higher than that recorded for nine sand dunes of the southern and northern coast of Bahia (Dias and Rocha 2014, *n* = 4–11), mountainous forests in southern Bahia (Rojas-Padilla et al. 2020, *n* = 7), fragments of ombrophilous forest of southeast Bahia (Dias et al. 2014b, *n* = 3 or 4), and the semideciduous seasonal forest of southeast Bahia (Souza-Costa et al. 2020, *n* = 6). Although the richness estimators revealed that there are lizard species that have not yet been sampled in the Cruz das Almas assemblage, their values were low, indicating that there may be few species. Additionally, the dissimilarity between the Cruz das Almas lizard assemblage and other assemblages from the Brazilian northeast, in particular those that formed the largest group, may be associated with the absence of some species, such as *Ameivulaocellifera*, *Enyaliusbibronii*, *Enyaliuscatenatus*, *Gymnodactylusdarwinii*, *Kentropyxcalcarata*, *Polychrusmarmoratus*, and *Tropidurussemitaeniatus*. These species are frequently found in inventory studies (see Roberto et al. 2017; Freitas et al. 2018; Mesquita et al. 2018), and their absence in the Cruz das Almas assemblage may have led to the pattern observed in our cluster.

Analysis of the species composition revealed two endemic species of the Atlantic Forest (*Phyllopezuslutzae* and *Dryadosauranordestina*), which corresponds to 14.9% of the lizard fauna recorded in the study (Tozetti et al. 2017). The presence of *P.lutzae*, *Diploglossuslessonae*, and *D.nordestina* can be considered unusual records for Cruz das Almas. *Phyllopezuslutzae* has the type locality Salvador, the capital of the Bahia State (Loveridge 1941), and since its description, the species has been reported for areas of Atlantic Forest in northeastern Brazil, often associated with restinga environments and using bromeliads as microhabitat (Vrcibradic et al. 2000; Albuquerque et al. 2019). In Cruz das Almas, the species was found inside the forests, using bromeliads and tree trunks, in syntopia with the congener *Phyllopezuspollicaris*. In addition, the species was also found on the edge of the forest patches, on the trunks of trees that had epiphytic bromeliads and isolated in pasture matrices. This record represents the distribution of the species outside the restinga environment, with insertion in interior forest patches, and highlights the need for efforts directed at expanding information on the spatial distribution and behaviour of the species.

The lizard *Diploglossuslessonae* can be found in areas of Atlantic Forest and Caatinga in northeast Brazil (Vanzolini et al. 1980). Although the presence of this species is well documented north of the São Francisco River, reports for Bahia are limited to the municipalities of Feira de Santana, Miguel Calmon and Santo Amaro (see Caldas et al. 2016), with the last report in 2009 (ca. 12 years ago). It is possible that the scarcity of information on *D.lessonae* records for the Bahia State is associated with the secretive habit and burrowing behaviour of the species (Vitt 1985), requiring greater field effort. In Cruz das Almas, *D.lessonae* was found in habitat with slightly compacted soil and higher density of leaf litter, which is perhaps a characteristic of the essential habitat for the presence of the species. Finally, *Dryadosauranordestina* is a species distributed in an area of Atlantic Forest of the Brazilian northeast. Although the occurrence of the species is well documented (Garda et al. 2014), information on populations in the State of Bahia is still scarce, and the species was included in the list of threatened fauna of the State of Bahia, in the vulnerable category (Sema 2017). A single individual from *D.nordestina* was found in Cruz das Almas, accessed through a pitfall trap in Mata de Cazuzinha. These data are different from the data of Lion et al. (2016), who pointed out *D.nordestina* as the most abundant species in small forest patches in the Rio Grande do Norte State. We believe that the increase in sample effort in more interior forest patches in eastern Bahia may reveal new records of occurrence of *Phyllopezuslutzae*, *D.lessonae*, and *D.nordestina*. Nevertheless, the encounter of *D.lessonae* and *D.nordestina* in the urban forest fragment highlights the importance of the preservation of the forest enclaves to maintain populations of these species.

For snakes, none of the species found in our study represents a new finding, as they are species that were previously registered in the State of Bahia and for the Atlantic Forest (Argôlo 2004; Hamdan and Lira-da-Silva 2012; Freitas 2014; Marques et al. 2016). Furthermore, *Bothropsleucurus* was the only species with a distribution endemic to the Atlantic Forest. A comparison of the richness of Cruz das Almas snakes with other assemblages of the Atlantic Forest revealed a similar value with several assemblages from north and south of the São Francisco River (Santana et al. 2008, *n* = 18; Marques et al. 2011, *n* = 15; Morato et al. 2011, *n* = 15) and south (Quintela et al. 2006, *n* = 16) and southeast Brazil (Araujo et al. 2010, *n* = 22; Vrcibradic et al. 2011, *n* = 19; Almeida-Gomes et al. 2012, *n* = 24). However, the richness value was lower than that observed in some snake assemblages in southeast Brazil (Condez et al. 2009, *n* = 46; Forlani et al. 2010, *n* = 48; Moura et al. 2012, *n* = 29) and north of the São Francisco River (Mesquita et al. 2018, *n* = 42). Within Bahia State, the snake richness of Cruz das Almas was lower than that registered on the northern coast (Marques et al. 2016, *n* = 50), Serra da Jibóia (Freitas et al. 2018, *n* = 37) and the southern and southeastern forests (Argôlo 2004, *n* = 61; Rojas-Padilla et al. 2020, *n* = 41) of the State. Moreover, the richness of snakes was greater than that obtained by Dias et al. (2014b) for four localities between the municipalities of Almadina, Floresta Azul, Ilhéus in southeast Bahia (5–8 species), and by Dias and Rocha (2014), who investigated the composition of snakes in nine localities of the sand dunes of Bahia (0–4 species). Nevertheless, the richness of Cruz das Almas snake species was similar to that obtained by Souza-Costa et al. (2020) for fragments of semideciduous seasonal forest in the Serras de Mandim and Azul in southwestern Bahia (13–18 species).

We believe that the difference in snake species richness between the assemblages of the Bahia State may be more associated with the sample design involved in the data collection than necessarily with a biological effect arising from the locality and study area. Dias et al. (2014b) performed a rapid inventory for data collection (12 days), which may have made it impossible to find seasonal or less abundant species, while Dias and Rocha (2014) only performed monitoring in restinga areas, not including other phyto-physiognomies, which may have reduced the sampling power only for species frequenting sand dunes. Similarly, the studies that showed high richness covered extensive areas, encompassing several municipalities and including different phyto-physiognomies (Argôlo 2004; Marques et al. 2016; Freitas et al. 2018). Thus, considering the existence of a directly proportional relationship between area vs. richness (Magurran 2004), it is possible that the size of the study area explains the variation in richness between studies and makes comparisons difficult.

Although we found a species richness similar to that presented by Souza-Costa et al. (2020), we noticed a difference in the composition of snake species, with the Cruz das Almas assemblage being dominated by species with a habit of living in open environments or being generalists in the use of habitat (Argôlo 2004), while there was a depletion of species more specialised to forested environments, typically of the genera *Corallus*, *Clelia*, *Dipsas*, and *Imantodes* and with previous records in the Serra da Jibóia (Freitas et al. 2018) and Serra do Timbó (Freitas et al. 2019), ca. 46 and 80 km from Cruz das Almas, respectively, and which we expected to be found in the studied area. The lack of more specialised species in forest environments may help explain why the Cruz das Almas snake assemblage was more similar to the Mata do Buraquinho and REBIO Guaribas assemblages. The former is inserted in an Atlantic Open Forest ecosystem and is characterised by being a less dense forest with opened canopy (Marques et al. 2021). The latter is inserted in an ecosystem of Stational Semidecidual Forest and “tabuleiros”, a type of vegetation of savanna similar to the Cerrado (Mesquita et al. 2018). Thus, these assemblages are subject to the dominance of species adapted to live in more open lands. As for the anurans, we believe that the absence of snakes specialised to forested environments that were expected to be found in the Cruz das Almas assemblage is an effect of anthropisation and habitat alteration, with the accentuated reduction and transformation of forested environments into cultivable areas and housing.

Finally, in this study, we report a new record of the water tiger *Trachemysdorbigni* for the Bahia State. The species is distributed throughout southern South America, in the countries of Argentina, Uruguay and Brazil, especially in the States of Rio Grande do Sul and Santa Catarina (Uetz et al. 2019). However, it is considered introduced into the Atlantic Forest (Tozetti et al. 2017) and has already been registered in the municipality of Salvador, capital of the Bahia State (Ecoa 2013). In Cruz das Almas, *T.dorbigni* was found wandering in a pasture area, with the presence of some sprawling residences. Thus, we do not rule out the possibility that the individual was being raised as a pet. Recent data have shown that Brazil has an intense reptile trade, which was enhanced by online shopping (Alves et al. 2019). In addition, the Recôncavo Baiano has a strong local trade of reptiles, with snakes being the main group traded (Macedo 2018). For Sy (2015) breeding reptiles as pets can promote the entry of exotic animals into ecosystems, and this is an extremely harmful phenomenon for native biota. Although we have no evidence that the presence of *T.dorbigni* in the studied assemblages comes from the trade of wild animals, we warn about the growth of the activity in the Recôncavo Baiano and the potential environmental damage that this entails, especially for ecosystems that are already heavily impacted.

The two amphisbaenian species identified in our study are common and frequently recorded in the Atlantic Forest inventories from the Brazilian northeast (Couto-Ferreira et al. 2011; Freitas et al. 2018; Mesquita et al. 2018). Despite the fossorial habit of the group, the number of species recorded in our study was similar to that recorded in other studies in the Atlantic Forest (Santana et al. 2008; Roberto et al. 2017; Rojas-Padilla et al. 2020). Perhaps the long-term fieldwork and the use of different methods of data collection have been important to record these organisms. Finally, the present study shows new data about the species composition of the herpetofauna in the Atlantic Forest of the east of Bahia, which helps to fill the information gap about the herpetofauna in unprotected areas. We highlight the prevalence of generalist species, typically associated with open lands and the presence of the threatened lizard *Dryadosauranordestina*, as well as the invasive turtle *Trachemysdorbigni* in forest patches. This information can be helpful for characterising the fauna of this region and the factors involved in determining the composition of amphibian and reptile species in other assemblages in the Atlantic Forest of northeast Brazil.

## References

[B1] Albuquerque PRA Morais MSR Moura PTS Santos WNS Costa RMT Delfim FR Pontes BES (2019) *Phyllopezuslutzae* (Loveridge, 1941) (Squamata, Phyllodactylidae): new records from the Brazilian state of Paraíba. Check List 15: 49–53. 10.15560/15.1.49

[B2] Almeida-Gomes M Siqueira CC Borges-Junior VNT Vrcibradic D Fusinatto LA Rocha CFD (2014) Herpetofauna of the Reserva Ecológica de Guapiaçu (REGUA) and its surrounding areas, in the state of Rio de Janeiro, Brazil. Biota Neotropica 14: 1–15. 10.1590/1676-0603007813

[B3] Almeida-Gomes M Rocha CFD (2014) Landscape connectivity may explain anuran species distribution in an Atlantic forest fragmented area. Landscape Ecology 29: 29–40. 10.1007/s10980-013-9898-5

[B4] Alves RRN Monielly B Araújo C Policarpo IS Pereira HM Borges AKM Vieira WLS Vasconcellos A (2019) Keeping reptiles as pets in Brazil: ethnozoological and conservation aspects. Journal for Nature Conservation 49: 9–21. 10.1016/j.jnc.2019.02.002

[B5] Andrade FS Haga IA Lyra ML Leite FSF Kwet A Haddad CFB Toledo LF Giaretta AA (2018) A new species of *Pseudopaludicola* Miranda-Ribeiro (Anura: Leptodactylidae: Leiuperinae) from eastern Brazil, with novel data on the advertisement call of *Pseudopaludicolafalcipes* (Hensel). Zootaxa 4433: 71–100. 10.11646/zootaxa.4433.1.4 30313239

[B6] Araujo CO, Condez TH, Bovo RP, Centeno FC, Luiz AM (201) Amphibians and reptiles of the Parque Estadual Turístico do Alto Ribeira (PETAR), SP: an Atlantic Forest remnant of Southeastern Brazil. Biota Neotropica 10: 257–274. 10.1590/S1676-06032010000400031

[B7] Argôlo AJS (2004) As serpentes dos Cacauais do Sudeste da Bahia. Editus, Ilhéus.

[B8] Armstrong CG Conte CE (2010) Taxocenose de anuros (Amphibia: Anura) em uma área de Floresta Ombrófila Densa no Sul do Brasil. Biota Neotropica 10: 39–46. 10.1590/S1676-06032010000100003

[B9] Azevedo PO (2011) Recôncavo: território, urbanização e arquitetura. In: Caroso C Tavares F Pereira C (Eds) Baía de Todos os Santos: aspectos humanos.EDUFBA, Salvador, 205–252.

[B10] Bastazini CV Munduruca JFV Rocha PLB Napoli MF (2007) Which environmental variables better explain changes in anuran community composition? a case study in the restinga of Mata de São João, Bahia, Brazil. Herpetologica 63: 459–471. 10.1655/0018-0831(2007)63[459:WEVBEC]2.0.CO;2

[B11] Bertoluci J Canelas MAS Eisemberg CC Palmuti CFS Montingelli GG (2009) Herpetofauna da Estação Ambiental de Peti, um fragmento de Mata Atlântica do estado de Minas Gerais, sudeste do Brasil. Biota Neotropica 9: 147–155. 10.1590/S1676-06032009000100017

[B12] Brazão JEM Araújo AP (1981) Vegetação. As regiões fitoecológicas, sua natureza e seus recursos econômicos. Estudo fitogeográfico. In: Brasil (Ed.) Folha SD.24 Salvador. Geologia, Geomorfologia, Pedologia, Vegetação, Uso Potencial da Terra. Ministério das Minas e Energia. Projeto RADAMBRASIL, Rio de Janeiro.

[B13] Caldas FLS Santana DO Faria RG Bocchiglieri A Mesquita DO (2016) *Diploglossuslessonae* Peracca, 1890 (Squamata: Anguidae): new records from northeast Brazil and notes on distribution. Check List 12: 1–5. 10.15560/12.5.1982

[B14] Camurugi F Lima TM Mercês EA Juncá FA (2010) Anurans of the Reserva Ecológica da Michelin, municipality of Igrapiúna, State of Bahia, Brazil. Biota Neotropica 10: 305–312. 10.1590/S1676-06032010000200032

[B15] Cicchi PJP Serafim H Sena MA Centeno FC Jim J (2009) Herpetofauna em uma área de Floresta Atlântica na Ilha Anchieta, município de Ubatuba, sudeste do Brasil. Biota Neotropica 9: 201–212. 10.1590/S1676-06032009000200019

[B16] Colombo P Kindel A Vinciprova G Krause L (2008) Composição e ameaças à conservação dos anfíbios anuros do Parque Estadual de Itapeva, município de Torres, Rio Grande do Sul, Brasil. Biota Neotropica 8: 229–240. 10.1590/S1676-06032008000300020

[B17] Colwell RK (2013) EstimateS: statistical estimation of species richness and shared species from samples. http://viceroy.eeb.uconn.edu/estimates/ [last accessed 1 August 2021]

[B18] Condez TH Sawaya RJ Dixo M (2009) Herpetofauna dos remanescentes de Mata Atlântica da região de Tapiraí e Piedade SP, sudeste do Brasil. Biota Neotropica 9: 157–185. 10.1590/S1676-06032009000100018

[B19] Costa HC Bérnils RS (2018) Répteis do Brasil e suas Unidades Federativas: lista de espécies. Herpetologia Brasileira 8: 11–57.

[B20] Couto-Ferreira D Tinôco MS Oliveira MLT Browne-Ribeiro HC Fazolato CP Silva RM Barreto GS Dias MA (2011) Restinga lizards (Reptilia: Squamata) at the Imbassaí Preserve on the northern coast of Bahia, Brazil. Journal of Threatened Taxa 3: 1990–2000. 10.11609/JoTT.o2800.1990-2000

[B21] Cruz CAG Napoli MF (2010) A new species of smooth horned frog, genus *Proceratophrys* Miranda-Ribeiro (Amphibia: Anura: Cycloramphidae), from the Atlantic rainforest of Eastern Bahia, Brazil. Zootaxa 2660: 57–67. 10.11646/zootaxa.2660.1.5

[B22] Cruz CAG Napoli MF Fonseca PM (2008) A new species of *Phasmahyla* Cruz, 1990 (Anura: Hylidae) from the State of Bahia, Brazil. South American Journal of Herpetology 3: 187–195. 10.2994/1808-9798-3.3.187

[B23] Dias EJR Rocha CFD (2014) Habitat structural effect on squamata fauna of the restinga ecosystem in Northeastern Brazil. Anais da Acadêmica Brassileira de Ciência 86: 359–371. 10.1590/0001-3765201420130006 24676173

[B24] Dias IR Medeiros TT Nova MFV Solé M (2014a) Amphibians of Serra Bonita, southern Bahia: a new hotpoint within Brazil’s Atlantic Forest hotspot. Zookeys 449: 105–130. 10.3897/zookeys.449.7494 PMC4233400 25408616

[B25] Dias IR Mira-Mendes CV Solé M (2014b) Rapid inventory of herpetofauna at the APA (Environmental Protection Area) of the Lagoa Encantada and Rio Almada, Southern Bahia, Brazil. Herpetology Notes 7: 627–637.

[B26] Dixo M Verdade VK (2006) Herpetofauna de serrapilheira da Reserva Florestal de Morro Grande, Cotia (SP). Biota Neotropica 6: 1–20. 10.1590/S1676-06032006000200009

[B27] Duellman WE Trueb L (1994) Biology of Amphibians. Johns Hopkins University Press, Baltimore.

[B28] Ecoa (2013) Animais e plantas do Parque Metropolitano de Pituaçu: lista de espécies. Centro de Ecologia e Conservação Animal.

[B29] Feio RN Ferreira PL (2005) Anfíbios de dois fragmentos de Mata Atlântica no município de Rio Novo, Minas Gerais. Revista Brasileira de Zoociências 7: 121–128.

[B30] Forlani MC Bernardo PH Haddad CFB Zaher H (2010) Herpetofauna do Parque Estadual Carlos Botelho, São Paulo, Brasil. Biota Neotropica 10: 265–309. 10.1590/S1676-06032010000300028

[B31] Freitas MA (2014) Squamate reptiles of the Atlantic Forest of northern Bahia, Brazil. Check List 10: 1020–1030. 10.15560/10.5.1020

[B32] Freitas MA Abegg AD Dias IR Moraes EPF (2018) Herpetofauna from Serra da Jibóia, an Atlantic Rainforest remnant in the state of Bahia, northeastern Brazil. Herpetology Notes 11: 59–72.

[B33] Freitas MA Colli GR Entiauspe-Neto OM Trinchão L Araújo D Lima TO França DPF Gaiga R Dias P (2016a) Snakes of Cerrado localities in western Bahia, Brazil. Check List 12: 1–10.

[B34] Freitas MA Entiauspe-Neto OM Lima TO Silva-Neto JS Araújo D Silva JMS (2016b) Snakes of Juazeiro, Bahia, middle of São Francisco River, Brazil. Boletim do Museu Biológico Mello Leitão 38: 331–345.

[B35] Freitas MA Silva TFS Fonseca PM Hamdan B Filadelfo T Abegg AD (2019) Herpetofauna of Serra do Timbó, an Atlantic Forest remnant in Bahia State, northeastern Brazil. Herpetology Notes 12: 245–260.

[B36] Freitas MA Veríssimo D Uhlig V (2012) Squamate reptiles of the central Chapada Diamantina, with a focus on the municipality of Mucugê, state of Bahia, Brazil. Check List 8: 16–22. 10.15560/8.1.016

[B37] Garda AA Costa TB Santos-Silva CR Mesquita DO Faria RG Conceição BM Silva IRS Ferreira AS Rocha SM Palmeira CNS Rodrigues R Ferrari SF Torquato S (2013) Herpetofauna of protected areas in the Caatinga In: Raso da Catarina Ecological Station (Bahia, Brazil). Check List 9: 405–414. 10.15560/9.2.405

[B38] Garda AA Medeiros PHS Lion MB Brito MRM Vieira GHC Mesquita DO (2014) Autoecology of *Dryadosauranordestina* (Squamata: Gymnophthalmidae) from Atlantic forest fragments in Northeastern Brazil. Zoologia 31: 418–425. 10.1590/S1984-46702014000500002

[B39] Gondim-Silva FAT Andrade ARS Abreu RO Nascimento JS Corrêa GP Menezes L Trevisan CC Camargo SS Napoli MF (2016) Composition and diversity of anurans in the Restinga of the Conde municipality, Northern coast of the state of Bahia, Northeastern Brazil. Biota Neotropica 16: 1–16. 10.1590/1676-0611-BN-2016-0157

[B40] Haddad CFB Toledo LF Prado CPA Loebmann D Gasparini JL Sazima I (2013) Guia de Anfíbios da Mata Atlântica: diversidade e biologia. Anolisbooks, São Paulo.

[B41] Hamdan B Lira-da-Silva RM (2012) The snakes of Bahia state, northeastern Brazil: species richness, composition and biogeographical notes. Salamandra 48: 31–50.

[B42] Heyer WR Donnelly MA McDiarmid RW Hayek LC Foster MS (1994) Measuring and monitoring biological diversity: standard methods for amphibians. Smithsonian Institution Press, Washington.

[B43] ICMBio (2018) Livro Vermelho da Fauna Brasileira Ameaçada de Extinção. Volume 1. ICMBio, Brasília.

[B44] Juncá FA (2006) Diversidade e uso de hábitat por anfíbios anuros em duas localidades de Mata Atlântica, no norte do estado da Bahia. Biota Neotropica 6: 1–17. 10.1590/S1676-06032006000200018

[B45] Lantyer-Silva ASF Siqueira-Júnior S Zina J (2013) Checklist of amphibians in a transitional area between the Caatinga and the Atlantic Forest, central-southern Bahia, Brazil. Check List 9: 725–732. 10.15560/9.4.725

[B46] Leite AK Oliveira MLT Dias MA Tinôco MS (2019) Species composition and richness of the herpetofauna of the semiarid environment of Nordestina, in northeastern Bahia, Brazil. Biotemas 32: 63–78. 10.5007/2175-7925.2019v32n4p63

[B47] Leite FSF Eterovick PC Juncá FA (2008) Status do conhecimento, endemismo e conservação de anfíbios anuros da Cadeia do Espinhaço, Brasil. Megadiversidade 4: 158–176.

[B48] Lion MB Garda AA Santana DJ Fonseca CR (2016) The conservation value of small fragments for Atlantic Forest reptiles. Biotropica 48: 265–275. 10.1111/btp.12277

[B49] Loveridge A (1941) *Bogertialutzae* - a new genus and species of gecko from Bahia, Brazil. Proceedings of the Biological Society of Washington 54: 195–196.

[B50] Lucas EM Marocco JC (2011) Anurofauna (Amphibia, Anura) em um remanescente de Floresta Ombrófila Mista no Estado de Santa Catarina, Sul do Brasil. Biota Neotropica 11: 377–384. 10.1590/S1676-06032011000100035

[B51] Macedo DS (2018) Etno-herpetologia no recôncavo baiano: perspectivas e consequências da criação de répteis. Monography. Universidade Federal do Recôncavo da Bahia, Cruz das Almas.

[B52] Magalhães FM Laranjeiras DO Costa TB Juncá FA Mesquita DO Röhr DL Silva WP Vieira GHC Garda AA (2015) Herpetofauna of protected areas in the Caatinga IV: Chapada Diamantina National Park, Bahia, Brazil. Herpetology Notes 8: 243–261.

[B53] Magalhães FM Dantas AKBP Brito MRM Medeiros PHS Oliveira AF Pereira TCSO Queiroz MHC Santana DJ Silva WP Garda AA (2013) Anurans from an Atlantic Forest-Caatinga ecotone in Rio Grande do Norte State, Brazil. Herpetology Notes 6: 1–10.

[B54] Magurran AE (2004) Measuring Biological Diversity. Blackwell, Oxford. 10.1016/j.cub.2021.07.049 34637726

[B55] Marques MCM Trindade W Bohn A Grelle CEV (2021) The Atlantic Forest: an introduction to the megadiverse forest of South America. In: Marques MCM Grelle CEV (Eds) The Atlantic Forest: history, biodiversity, threats and opportunities of the mega-diverse forest.Springer, Cham, 3–22.

[B56] Marques R Mebert K Fonseca E Rödder D Solé M Tinôco MS (2016) Composition and natural history notes of the coastal snake assemblage from Northern Bahia, Brazil. Zookeys 611: 93–142. 10.3897/zookeys.611.9529 PMC4992808 27594800

[B57] Marques R Tinôco MS Couto-Ferreira D Fazolato CP Browne-Ribeiro HC Travassos MLO Dias MA Mota JVL (2011) Reserva Imbassaí Restinga: inventory of snakes on the northern coast of Bahia, Brazil. Journal of Threatened Taxa 3: 2184–2191. 10.11609/JoTT.o2812.2184-91

[B58] Mesquita DO Alves BCF Pedro CKB Laranjeiras DO Caldas FLS Pedrosa IMMC Rodrigues JB Drummond LO Cavalcanti LBQ Wachlevski M Nogueira-Costa P França RC França FGR (2018) Herpetofauna in two habitat types (tabuleiros and Stational Semidecidual Forest) in the Reserva Biológica Guaribas, northeastern Brazil. Herpetology Notes 11: 455–474.

[B59] Mira-Mendes CV Ruas DS Oliveira RM Castro IM Dias IR Baumgarten JE Juncá FA Solé M (2018) Amphibians of the Reserva Ecológica Michelin: a high diversity site in the lowland Atlantic Forest of southern Bahia, Brazil. Zookeys 753: 1–21. 10.3897/zookeys.753.21438 PMC5934352 29731680

[B60] Mittermeier RA Gil PR Hoffmann M Pilgrim J Brooks T Mittermeier CG Lamoreux J Fonseca GAB (2004) Hotspots revisited. CEMEX, Mexico City.

[B61] Mônico AT Clemente-Carvalho RBG Lopes SR Peloso PLV (2017) Anfíbios anuros de brejos e lagoas de São Roque do Canaã, Espírito Santo, Sudeste do Brasil. Papéis Avulsos de Zoologia 57: 197–206. 10.11606/0031-1049.2017.57.16

[B62] Morato SAA Lima AMX Staut DCP Faria RG Souza-Alves JP Gouveia SF Scupino MRC Gomes R Silva MJ (2011) Amphibians and Reptiles of the Refúgio de Vida Silvestre Mata do Junco, municipality of Capela, state of Sergipe, northeastern Brazil. Check List 7: 756–762. 10.15560/11015

[B63] Moura MR Argôlo AJS Costa HC (2017) Historical and contemporary correlates of snake biogeographical subregions in the Atlantic Forest hotspot. Journal of Biogeography 44: 640–650. 10.1111/jbi.12900

[B64] Moura MR Motta AP Fernandes VD Feio RN (2012) Herpetofauna da Serra do Brigadeiro, um remanescente de Mata Atlântica em Minas Gerais, Sudeste do Brasil. Biota Neotropica 12: 209–235. 10.1590/S1676-06032012000100017

[B65] Napoli MF Silva LM Abreu RO (2017) Anfíbios. In: Nunes JMC Matos MRB (Eds) Litoral Norte da Bahia: caracterização ambiental, biodiversidade e conservação.EDUFBA, Salvador, 357–392.

[B66] Narvaes P Bertoluci J Rodrigues MT (2009) Composição, uso de hábitat e estações reprodutivas das espécies de anuros da floresta de restinga da Estação Ecológica Juréia-Itatins, sudeste do Brasil. Biota Neotropica 9: 117–123. 10.1590/S1676-06032009000200011

[B67] Palmeira CNS Gonçalves U (2015) Anurofauna de uma localidade na Mata atlântica setentrional, Alagoas, Brasil. Boletim do Museu de Biologia Mello Leitão 37: 141–163.

[B68] Quintela FM Loebmann D Gianuca NM (2006) Répteis continentais do município de Rio Grande, Rio Grande do Sul, Brasil. Biociências 14: 180–188.

[B69] Rezende CL Scarano FR Assad ED Joly CA Metzger JP Strassburg BBN Tabarelli M Fonseca GA Mittermeier RA (2018) From hotspot to hopespot: an opportunity for the brazilian Atlantic Forest. Perspectives in Ecology and Conservation 16: 208–214. 10.1016/j.pecon.2018.10.002

[B70] Roberto IJ Oliveira CR Araújo-Filho JA Oliveira HF Ávila RW (2017) The herpetofauna of the Serra do Urubu mountain range: a key biodiversity area for conservation in the brazilian Atlantic Forest. Papéis Avulsos de Zoologia 57: 347–373. 10.11606/0031-1049.2017.57.27

[B71] Rocha CFD Hatano FH Vrcibradic D Van Sluys M (2008) Frog species richness, composition and β-diversity in coastal Brazilian restinga habitats. Brazilian Journal of Biology 68: 101–107. 10.1590/S1519-69842008000100014 18470383

[B72] Rodrigues MT (1996) Lizards, snakes, and amphisbaenians from the Quaternary sand dunes of the middle Rio Sao Francisco, Bahia, Brazil. Journal of Herpetology 30: 513–523. 10.2307/1565694

[B73] Rodrigues MT (2005) Conservação dos répteis brasileiros: os desafios para um país megadiverso. Megadiversidade 1: 87–94.

[B74] Rojas-Padilla O Menezes VQ Dias IR Argôlo AJS Solé M Orrico VGD (2020) Amphibians and reptiles of Parque Nacional da Serra das Lontras: an important center of endemism within the Atlantic Forest in southern Bahia, Brazil. Zookeys 1002: 159–185. 10.3897/zookeys.1002.53988 33363431 PMC7746662

[B75] Rossa-Feres DC Garey MV Caramaschi U Napoli MF Nomura F Bispo AA Brasileiro CA Thomé MTC Sawaya RJ Conte CE Cruz CAG Nascimento LB Gasparini JL Almeida AP Haddad CFB (2017) Anfíbios da Mata Atlântica: Lista de espécies, histórico dos estudos, biologia e conservação. In: Monteiro-Filho ELA Conte CE (Eds) Revisões em Zoologia: Mata Atlântica.Editora UFPR, Curitiba, 237–314.

[B76] Sansone L (2011) Um contraponto baiano de açúcar e petróleo: mercadorias globais, identidades globais? In: Caroso C Tavares F Pereira C (Eds) Baía de Todos os Santos: aspectos humanos.EDUFBA, Salvador, 351–375.

[B77] Santana DJ São Pedro VA Hote PS Roberti HM Sant’Anna AC Figueiredo-de-Andrade CA Feio RN (2010) Anurans in the region of the High Muriaé River, state of Minas Gerais, Brazil. Herpetology Notes 3: 1–10.

[B78] Santana GG Vieira WLS Pereira-Filho GA Delfim FR Lima YCC Vieira KS (2008) Herpetofauna em um fragmento de Floresta Atlântica no Estado da Paraíba. Biotemas 21: 75–84. 10.5007/2175-7925.2008v21n1p75

[B79] Segalla MV Caramaschi U Cruz CAG Garcia PCA Grant T Haddad CFB Santana DJ Toledo LF Langone JA (2019) Lista de espécies brasileiras. Herpetologia Brasileira 8: 65–96.

[B80] Sema (2017) Lista Oficial das Espécies da Fauna Ameaçadas de Extinção do Estado da Bahia. Portaria SEMA n° 37.

[B81] Seplan (2015) Zoneamento Ecológico-Econômico do Estado da Bahia. Secretaria do Planejamento, Salvador.

[B82] Silva JMC Casteleti CHM (2003) Status of the biodiversity of the Atlantic Forest of Brazil. In: Galindo-Leal C Câmara IG (Eds) The Atlantic forest of South America: biodiversity status, threats, and outlook.CABS and Island Press, Washington, 43–59.

[B83] Silva TSM Coelho-Filho MA Coelho EF (2016) Boletim meteorológico da estação convencional de Cruz das Almas, BA: variabilidade e tendências climáticas. Embrapa Mandioca e Fruticultura, Cruz das Almas.

[B84] Silvano DL Segalla MV (2005) Conservação de anfíbios no Brasil. Megadiversidade 1: 79–86.

[B85] Souza-Costa CA Mira-Mendes CV Dias IR Silva KB Argôlo AJS Solé M (2020) Squamate reptiles from seasonal semi-deciduous forest remnants in southwestern Bahia, Brazil. Bonn Zoological Bulletin 69: 85–94.

[B86] Sy EY (2015) Checklist of exotic species in the Philippine pet trade, II. Reptiles. Journal of Nature Studies 14: 66–93.

[B87] Telles FBS Menezes VA Maia-Carneiro T Dorigo TA Winck GR Rocha CFD (2012) Anurans from the “Restinga” of Parque Natural Municipal de Grumari, state of Rio de Janeiro, southeastern Brazil. Check List 8: 1267–1273. 10.15560/8.6.1267

[B88] Tinôco MS Browne-Ribeiro HC Souza-Alves JP (2007) Snake communities and Atlantic Forest conservation in Brazil. Solitaire 18: 8–9.

[B89] Tozetti AM Sawaya RJ Molina FB Bérnils RS Barbo FE Leite JCM Borges-Martins M Recoder RS Teixeira-Junior M Argôlo AJS Morato SAA Rodrigues MT (2017) Répteis. In: Monteiro-Filho ELA Conte CE (Eds) Revisões em Zoologia: Mata Atlântica.Editora UFPR, Curitiba, 315–364.

[B90] Uetz P Freed P Hošek J (2019) The Reptile Database. http://www.reptile-database.org/ [last acessed 1 August 2021]

[B91] Valdujo PH Recoder RS Vasconcellos MM Portella AS (2009) Amphibia, Anura, São Desidério, western Bahia uplands, northeastern Brazil. Check List 5: 903–911. 10.15560/5.4.903

[B92] Vanzolini PE Ramos-Costa AMM Vitt LJ (1980) Répteis das Caatingas. Academia Brasileira de Ciências, Rio de Janeiro.

[B93] Vasconcelos TS Prado VHM Silva FR Haddad CFB (2014) Biogeographic distribution patterns and their correlates in the diverse frog fauna of the Atlantic Forest hotspot. PLoS ONE 9: 1–9. 10.1371/journal.pone.0104130 PMC4139199 25140882

[B94] Vitt LJ (1985) On the biology of the little known anguid lizard, *Diploglossuslessonae* in Northeast Brazil. Papéis Avulsos de Zoologia 36: 69–76.

[B95] Vrcibradic D Hatano FH Rocha CFD Sluys MVS (2000) Geographic distribution. *Bogertialutzae*. Herpetologial Review 31: 112.

[B96] Vrcibradic D Rocha CFD Kiefer MC Hatano FH Fontes AF Almeida-Gomes M Siqueira CC Pontes JAL Borges-Junior VNT Gil LO Klaion T Rubião ECN Van Sluys M (2011) Herpetofauna, Estação Ecológica Estadual do Paraíso, state of Rio de Janeiro, southeastern Brazil. Check List 7: 745–749. 10.15560/11013 21971595

[B97] Xavier AL Napoli MF (2011) Contribution of environmental variables to anuran community structure in the Caatinga Domain of Brazil. Phyllomedusa 10: 45–64.

[B98] Zanella N Paula A Guaragni SA Machado LS (2013) Herpetofauna do Parque Natural Municipal de Sertão, Rio Grande do Sul, Brasil. Biota Neotropica 13: 290–298. 10.1590/S1676-06032013000400026

[B99] Zina J Ennser J Pinheiro SCP Haddad CFB Toledo LF (2007) Taxocenose de anuros de uma mata semidecídua do interior do Estado de São Paulo e comparações com outras taxocenoses do Estado, sudeste do Brasil. Biota Neotropica 7: 49–57. 10.1590/S1676-06032007000200005

